# Discrete shear band plasticity through dislocation activities in body-centered cubic tungsten nanowires

**DOI:** 10.1038/s41598-018-23015-z

**Published:** 2018-03-15

**Authors:** Jiangwei Wang, Yanming Wang, Wei Cai, Jixue Li, Ze Zhang, Scott X. Mao

**Affiliations:** 1 Center of Electron Microscopy and State Key Laboratory of Silicon Materials, School of Materials Science and Engineering, Zhejiang University, Hangzhou, 310027 China; 2 Department of Mechanical Engineering and Materials Science, University of Pittsburgh, Pittsburgh, Pennsylvania 15261 USA; 3 Research Laboratory of Electronics, Massachusetts Institute of Technology, Cambridge, Massachusetts 02139 USA; 4 Department of Mechanical Engineering, Stanford University, Stanford, California 94305 USA

## Abstract

Shear band in metallic crystals is localized deformation with high dislocation density, which is often observed in nanopillar deformation experiments. The shear band dynamics coupled with dislocation activities, however, remains unclear. Here, we investigate the dynamic processes of dislocation and shear band in body-centered cubic (BCC) tungsten nanowires via an integrated approach of *in situ* nanomechanical testing and atomistic simulation. We find a strong effect of surface orientation on dislocation nucleation in tungsten nanowires, in which {111} surfaces act as favorite sites under high strain. While dislocation activities in a localized region give rise to an initially thin shear band, self-catalyzed stress concentration and dislocation nucleation at shear band interfaces cause a discrete thickening of shear band. Our findings not only advance the current understanding of defect activities and deformation morphology of BCC nanowires, but also shed light on the deformation dynamics in other microscopic crystals where jerky motion of deformation band is observed.

## Introduction

Plastic deformation of crystals is controlled by the generation and propagation of lattice defects, most importantly, dislocations. As the crystal size scales down, the dynamic behavior of dislocations is significantly different from that in the bulk^1–3^. Currently, experimental and theoretical studies about the defect dynamics in small crystals are largely focused on face-centered cubic (FCC) metals and alloys^1–9^. In bulk FCC crystals, dislocations are usually generated by the Frank-Read sources, grain boundaries, or other pre-existing defects/interfaces^10,11^. At the microscopic length scale, the dominant dislocation generation mechanisms change to Frank-Read/single-arm source^3,4,12^ or surface source^1,5,6,13^, depending on the crystal size^14^. It was also shown that the preferred surface nucleation site and deformation mechanism in small FCC metals are strongly influenced by the surface step, side surface and cross-section shape of the samples^1,7,9,15,16^. However, much less is known about the surface effect on dislocation dynamics in microscopic crystals with other structures.

Body-centered cubic (BCC) metallic nanostructures hold great promise in novel micro-/nano- electromechanical systems (MEMS/NEMS), due to their high strength and excellent high-temperature performance. In small BCC crystals, dislocation activities usually dominate the plastic deformation at room temperature^4,17–22^. Nonetheless, the dynamic processes of deformation and failure of nanosized BCC metals remain poorly understood, especially at the atomic scale, primarily due to the technical challenges in fabricating and testing the nanosized BCC materials. Recently, enabled by an unique method of *in situ* welding, twinning-dominated deformation has been revealed in tungsten (W) nanowires with different orientations under room temperature and low strain rate^23^. An exception was the [112]-orientated nanowire, showing substantial plastic flow via dislocation and shear band activities under both tension and compression^23^. Such shear induced deformation bands were also frequently observed during the nanocompression of various metallic nanopillars, including both the FCC and BCC metals^18,24–28^; yet, the fundamental mechanism regarding shear band dynamics remains largely unknown, especially on how they are coupled with dislocation activities.

To clarify these uncertainties, here we study the deformation of [112]-W nanowires using *in situ* nanomechanical testing under high-resolution transmission electron microscopy (HRTEM) and atomistic simulations. We show that dislocation nucleation and shear band are coupled. The dislocation dipoles are nucleated as half dislocation loops, mostly from $(\mathop{1}\limits^{-}\mathop{1}\limits^{-}1)$ surfaces, which are preferred nucleation sites under high strain. Multiple dislocation dipoles are nucleated nearly simultaneously, forming an initial shear band. The stress concentration near the border of the shear band causes a preferential dislocation nucleation in this region, leading to discrete thickening of the shear band.

## Results

### Dislocation plasticity in [112]-W nanowires

**Figure 1:**
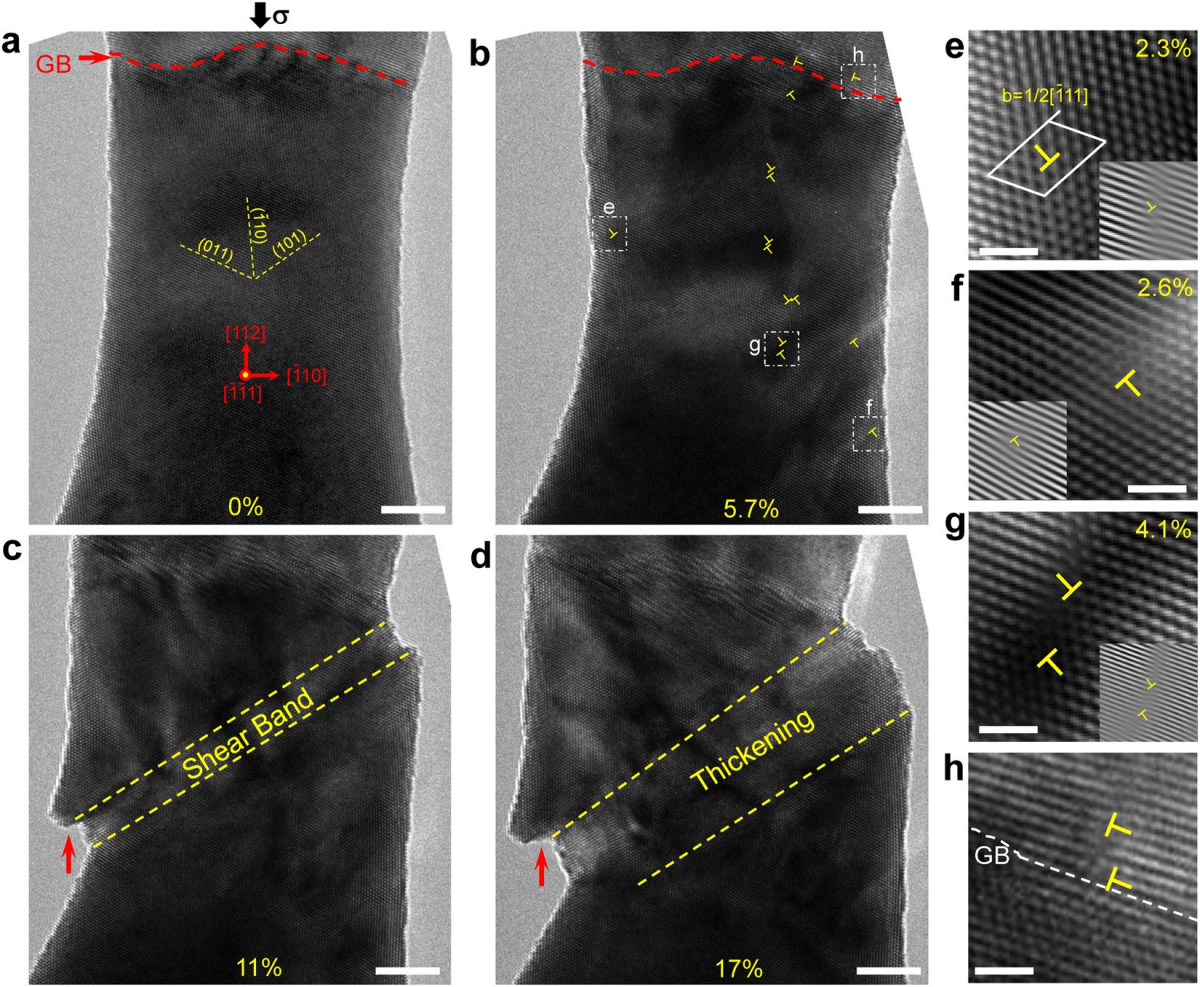
Dislocation and shear band mediated plasticity in a [112]-oriented W nanowire. (**a**) The morphology of pristine W nanowire without observable crystal defects. The nanowire diameter is about 21 nm. (**b**) Numerous dislocation activities are observed in the W nanowire after yielding, most of which are shown as dipoles. (**c**) Further deformation results in the formation of a shear band, (**d**) the thickening of which contributes to large plasticity. (**e**,**f**) Fourier-filtered HRTEM images showing the dislocations nucleated from the side surface under different compressive strains: (**e**) at the compressive strain of ~2.3% (compressive strain at the initial yielding point) and (**f**) at the compressive strain of ~2.6%. (**g**) Fourier-filtered HRTEM image showing the dislocation dipole nucleated from the viewing surface at the compressive strain of 4.0%. (**h**) HRTEM image showing the dislocations nucleated from the grain boundary and propagated into the upper grain. Scale bars are 5 nm in (**a**–**d**) and 1 nm in (**e**–**h**).

Since dislocation plasticity only occurs in W nanowires under $\langle 112\rangle$ loading^23^, here we will focus on the deformation of $\langle 112\rangle$-oriented W nanowires. Figure 1 shows an example of the dislocation and shear band mediated plasticity in a W bicrystal nanowire. Under compression, deformation mainly occurs in the large [112]-oriented grain of the bicrystal, denoted as the [112]-W nanowire. Before deformation, no lattice defect is observed in the [112]-orientated grain, suggesting the nature of perfect crystal after sample preparation (Fig. 1a). During deformation, the compression loading causes the strain accumulation inside the nanowire, which finally results in the yielding of nanowire via the heterogeneous nucleation of dislocations from free surface. The nucleation behavior of dislocation is affected by the deformation strain, with the favorable nucleation site changed from the side surface to the viewing surface with the increase of compressive strain (Fig. 1b,e–g and Supplementary Fig. S1). Specifically, at the low strain level, dislocations are mainly nucleated from multiple sites of side surface with a surface normal of $[\mathop{1}\limits^{-}10]$, which are identified to be 1/2 $[\mathop{1}\limits^{-}11]$-type mixed dislocations on (101) planes (often referred to as M111 dislocations)^29,30^, as shown by the dislocations nucleated at the compressive strain of ~2.3% (compressive strain at the initial yielding point) and ~2.6% in Fig. 1e,f, respectively; however, as the compressive strain further increases (high strain level), dislocations prefer to nucleate as dipoles close to the viewing surface with a surface normal of $[\mathop{1}\limits^{-}\mathop{1}\limits^{-}1]$ (Fig. 1b), as demonstrated by the dislocation dipole nucleated at the compressive strain of 4.0% in Fig. 1g; besides, it is noted that grain boundary in the bicrystal also acts as another effective dislocation source, and the dislocations emitted from the grain boundary can easily propagate into both grains after nucleation (Fig. 1b,h). As the nucleation events (from both the multiple surface sources and the grain boundary) proceed continually, dislocation density in the W nanowire increases significantly (Fig. 1b), which reaches about 2.6 × 10^16^ m^−2^ (calculated based on the number of dislocation lines crossing unit area in the HRTEM image) just before the sudden strain softening. The massive dislocation activities finally result in the sudden formation of a shear band at higher deformation strain, and its thickening provides large localized plasticity to accommodate the deformation (Fig. 1c,d). Accompanied with the shear band is the generation and enlargement of surface steps, suggesting the occurrence of significant dislocation slip at the interface between the shear band and the surrounding crystal (pointed out by the red arrow in Fig. 1c,d). The Fourier-filtered HRTEM image in Supplementary Fig. S2 also shows that there are plenty of dislocations near the shear band interfaces, while it is relatively clean inside the shear band. These facts indicate that the formation of shear band is an interface-controlled dislocation-mediated process. The shear band structure seen here in the [112]-W nanowire is similar to the deformation morphologies previously observed in the compression of both FCC and BCC nanopillars^18,19,24,25^, in which deformation bands were frequently observed, accompanied by significant stress drops.

### Dislocation nucleation mechanism

**Figure 2:**
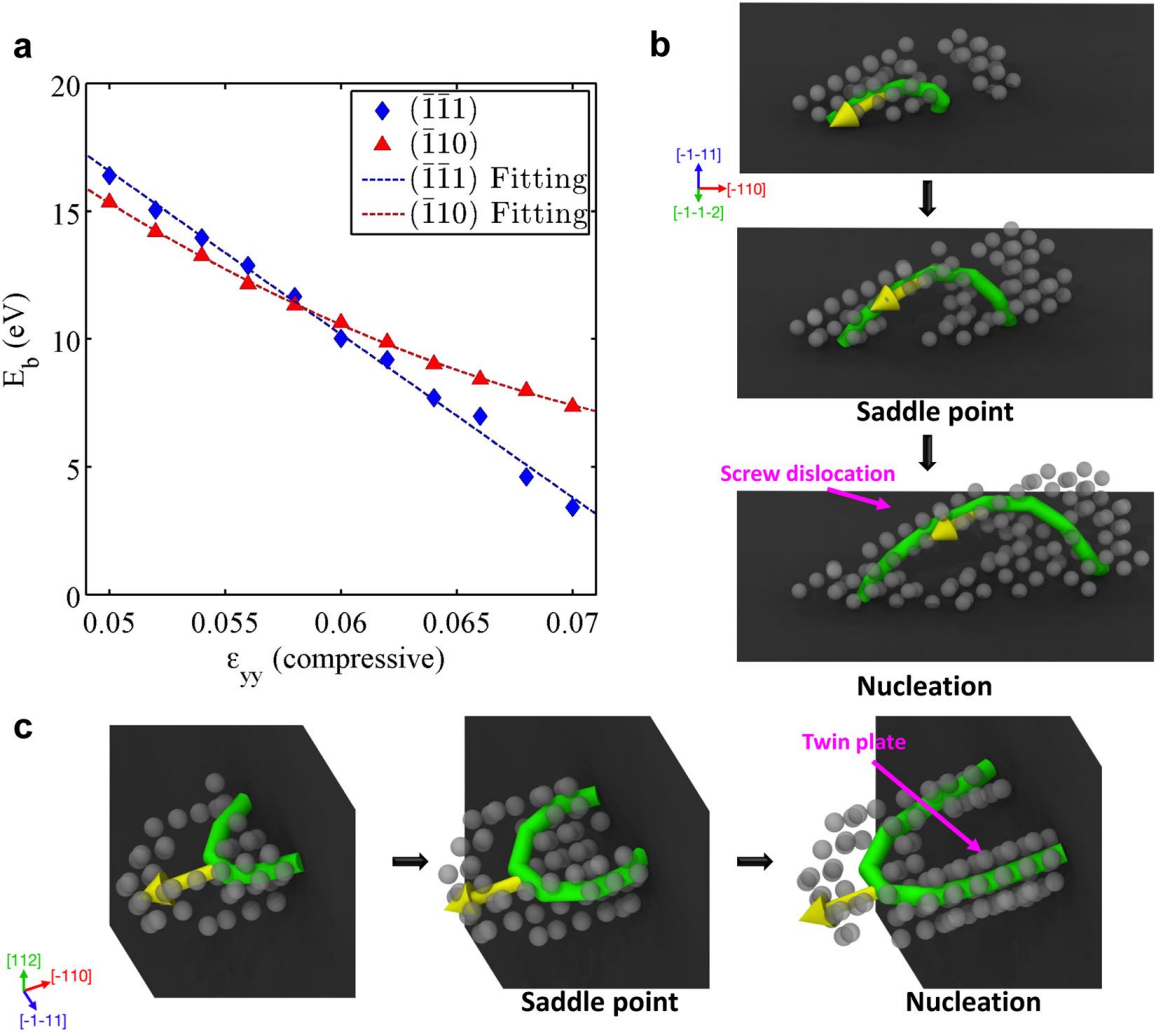
Atomistic simulations of the nucleation of dislocation loops from free surfaces in W. (**a**) Energy barriers for heterogeneous dislocation nucleation from the $(\mathop{1}\limits^{-}\mathop{1}\limits^{-}1)$ and $(\mathop{1}\limits^{-}10)$ surfaces in W as a function of strain. (**b**) Sequential snapshots showing atomic structure near the saddle point at a normal strain of ε_yy_ = 0.066 (compressive) for nucleation from the $(\mathop{1}\limits^{-}\mathop{1}\limits^{-}1)$ surface. (**c**) Sequential snapshots showing the atomic structure near the saddle point at normal strain ε_yy_ = 0.066 (compressive) for nucleation from the $(\mathop{1}\limits^{-}10)$ surface.

**Figure 3:**
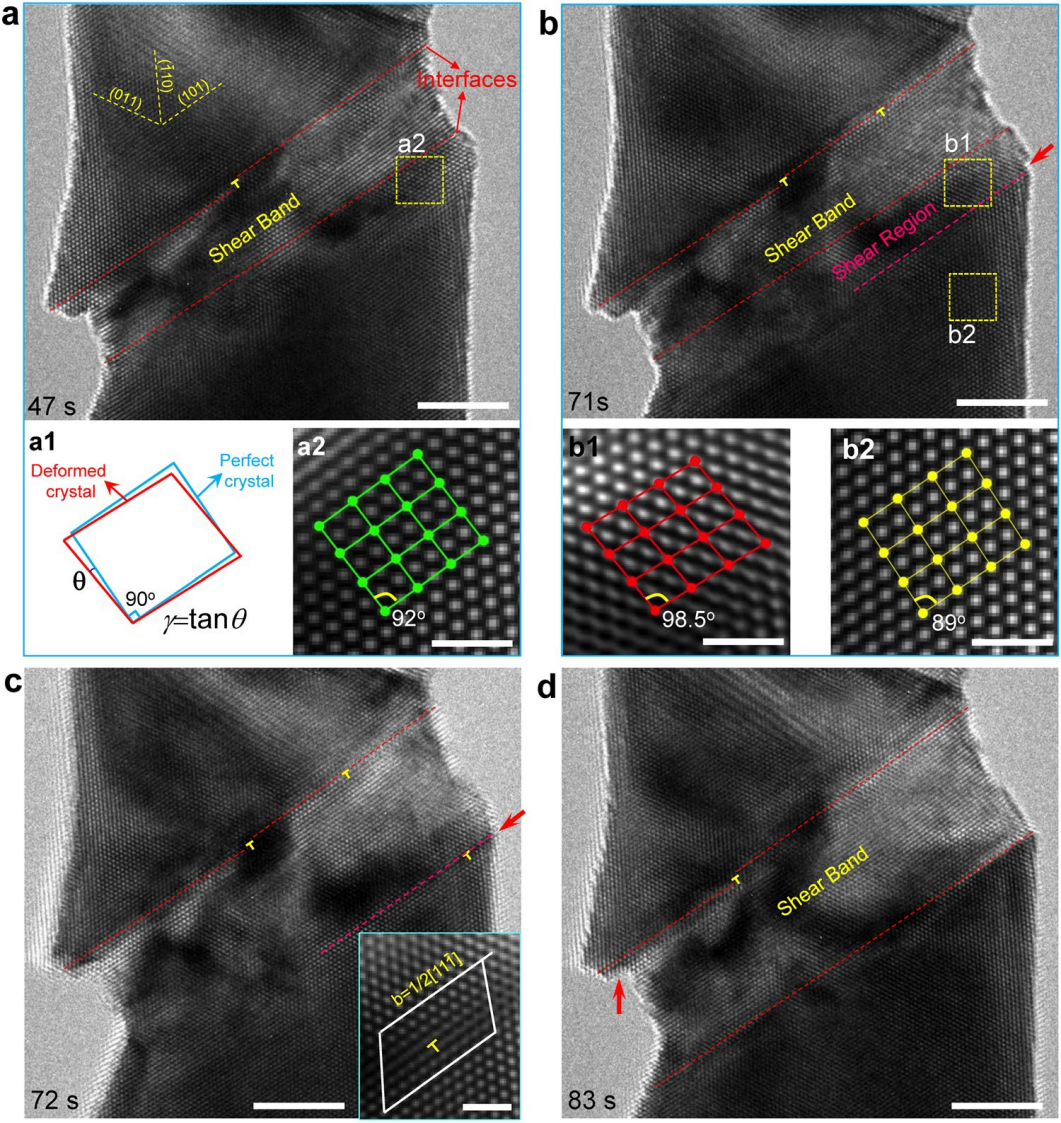
Thickening of shear band in a [112]-oriented W nanowire. (**a**) Initial morphology of the shear band with sharp interfaces and atomic-scale steps. (a1) Schematic of the calculation of lattice shear strain on the (101) slip plane. (a2) Shear deformation in the lattice outside of the shear band but near its interface. (**b**) Further compression results in a shear region with the lattice shear angle of 98.5° (b1) and surface steps (pointed by the red arrow in (**b**)). (b2) Negligible shear deformation occurs in the lattice away from the shear band interface. (**c**) A dislocation nucleates from the side surface at the shear region interface (pointed out by the red arrow) and propagates into the crystal. (**d**) The propagation of dislocations finally induces a new and stable shear band interface, leading to the thickening of shear band. The red arrow indicates the enlargement of surface steps at upper shear band interface. Scale bars are 5 nm in (**a**–**d**) and 1 nm in (a2), (b1-b2) and inset in (**c**).

Above observations indicate that the nucleation behavior of dislocations is affected by the strain level and surface structure. Due to the presence of nearby free surfaces, these dipoles nucleated at high strain level are likely the result of heterogeneous nucleation of half dislocation loops from the surface invisible in the HRTEM image, *i.e*. the $(\mathop{1}\limits^{-}\mathop{1}\limits^{-}1)$ surface in Fig. 1. The prevalence of dipoles away from the $(\mathop{1}\limits^{-}10)$ side surface in Fig. 1 suggests that dislocation nucleation is more favorable when the surface normal is closer to $[\mathop{1}\limits^{-}\mathop{1}\limits^{-}1]$ than $[\mathop{1}\limits^{-}10]$. This is consistent with the nucleation preference of dislocation to the screw orientation during the early stage of nucleation. Previous work in FCC gold nanowires has shown that the heterogeneous dislocation nucleation is favored at those surface locations where critical dislocation nucleus is mostly aligned in the screw orientation^9^. Once nucleated from $(\mathop{1}\limits^{-}\mathop{1}\limits^{-}1)$ surface, the screw dislocation embryo would grow into a half dislocation loop that possesses two edge segments (Fig. 2b), shown as a dislocation dipole under HRTEM. To investigate this in the BCC W nanowires, we have computed the energy barriers for dislocation nucleation from two surfaces, $(\mathop{1}\limits^{-}\mathop{1}\limits^{-}1)$ and $(\mathop{1}\limits^{-}10)$, which represent the nucleation from the viewing and side surfaces, respectively. The simulation details can be found in Methods. We note that the $(\mathop{1}\limits^{-}\mathop{1}\limits^{-}1)$ surface has a zigzag profile (involving three atomic layers), while the $(\mathop{1}\limits^{-}10)$ surface appears atomically flat (with a single layer), (Supplementary Fig. S3). The zigzag structure of $(\mathop{1}\limits^{-}\mathop{1}\limits^{-}1)$ surface could generate some local strain concentration at the surface facets during deformation^1^, favoring the dislocation nucleation. Simulation results show that for both surfaces, the energy barriers for dislocation nucleation drop dramatically as a function of the applied strain (Fig. 2a and Supplementary Fig. S). At high strains, the energy barrier is lower for the nucleation from $(\mathop{1}\limits^{-}\mathop{1}\limits^{-}1)$ surface than that from $(\mathop{1}\limits^{-}10)$ surface (Fig. 2a), which confirms our experimental observation that $(\mathop{1}\limits^{-}\mathop{1}\limits^{-}1)$ is the favored nucleation surface in BCC W nanowire under high strain level. However, the energy barrier for $(\mathop{1}\limits^{-}10)$ nucleation is competitive with that of $(\mathop{1}\limits^{-}\mathop{1}\limits^{-}1)$ nucleation at lower strains, explaining why we also see some nucleation events from the side surface upon the initial yielding of W nanowire. Figure 2b and Supplementary Fig. 5b shows that the dislocation nucleus from the $(\mathop{1}\limits^{-}\mathop{1}\limits^{-}1)$ surface contains an elongated screw segment that is able to cross-slip easily, which explains why two dislocations in some of the dipoles are located on adjacent glide planes (Supplementary Fig. S2) and why there are some atomic-scale ledges formed on the shear plane interface (Fig. 3). For the $(\mathop{1}\limits^{-}10)$ surface nucleation, the nucleus expands to a curved half dislocation loop under straining (Fig. 2c and Supplementary Fig. 5a), similar to the nucleation in FCC metals^9^, and the observed 1/2 $[\mathop{1}\limits^{-}11]$-type mixed dislocations emitted from the $(\mathop{1}\limits^{-}10)$ side surface (Fig. 1e,f) should be this type of half dislocation loop, where only one edge segment can be observed. These simulations show that the dislocation activities in [112]-W nanowires can be interpreted as the heterogeneous nucleation of half dislocation loops on {110} slip planes from the different surfaces, and the surface structure plays an important role in the dislocation nucleation of small BCC metals.

### Discrete thickening of the shear band

The shear band morphologies in metallic nanopillars were usually attributed to the dislocation slip^19,31^, although there has not been much discussion on how the shear band evolves dynamically. Here our atomic-scale observation shows that the shear band thickens in a discrete manner via a self-catalytic process of stress concentration and dislocation nucleation, distinct from the layer-by-layer thickening of deformation twinning in both FCC^7^ and BCC^23^ metals. Figure 3 show the dynamic thickening of a shear band in [112]-W nanowire. Initially, the shear band has sharp interfaces parallel to the (101) slip plane, with atomic-scale ledges on the interface, corresponding to isolated dislocations (Fig. 3a). However, further straining induces a region outside but adjacent to the shear band with significantly higher elastic shear strain, denoted as “shear region” in Fig. 3b. The calculation of resolved shear strain on the (101) slip plane from HRTEM images is shown in Fig. 3a,b. At time 47 s (after the initial formation of the shear band), the average resolved shear strain in region a2 is γ = tan (2°) = 0.035 (Inset a2 in Fig. 3a). However, at time 71 s (after the load is further increased), the resolved shear strain in the same region (now labeled as b1 in Fig. 3b) is γ = tan (8.5°) = 0.15, while the resolved shear strain in a region (b2 in Fig. 3b) far away from the shear band is much smaller, γ = tan (−1°) = −0.017. Here, the negative resolved shear strain in region b2 is likely caused by a small bending induced by the loading mechanism. We hypothesize that the enhanced resolved shear strain in the shear region adjacent to the shear band is caused by the non-symmetric geometry of the sample following the formation of the shear band. To test this hypothesis, we performed Finite Element Method (FEM) stress analysis of the W nanowire containing a shear band. Supplementary Fig. S6 shows that when the top surface of the nanowire moves rigidly downward, the resolved shear strain is everywhere positive with the region close to the shear band having a much higher magnitude. When the downward motion of the top surface is accompanied by a slight rotation, then the resolved shear strain in a region (region D in Supplementary Fig. S6d) far below the shear band can indeed turn negative, although the resolved shear strain near the shear band still has a large positive value.

Due to the greatly enhanced value of the resolved shear strain in the shear region (e.g. region b1 in Fig. 3b), dislocation nucleation event is expected to occur in this region when the load is further increased. As a dislocation nucleates from the side surface of the shear region (Fig. 3c), a new shear band interface is created by dislocation slip, which merges the whole shear region with the initial shear band, inducing the discrete thickening. That is, the lower interface of the shear band suddenly jumps by about 10 atomic layers (Fig. 3c,d), different from the layer-by-layer thickening of twinning^23^. These observations also indicate that in nanowire or nanopillar samples, the thickening of shear band proceeds through a self-catalytic process of stress concentration and dislocation nucleation near the shear band interface. The initial formation of shear band causes a deformation affected zone (i.e. shear region) adjacent to its interface, in which the high stress concentration catalyzes the nucleation of new dislocations. The dislocation propagation creates a new shear band interface and causes the whole shear region to merge into the shear band, resulting in a self-catalytic thickening. Consequently, the shear band interface does not move smoothly, but jumps forward in large steps, *i.e*. the discrete thickening. We suggest that this type of jerky motion of shear band interface may also occur in nanopillars of other crystal structures, in which similar shear-band induced geometries were widely observed^18,19,24,25,28^.

### Recoverable shear band in loading cycle

**Figure 4:**
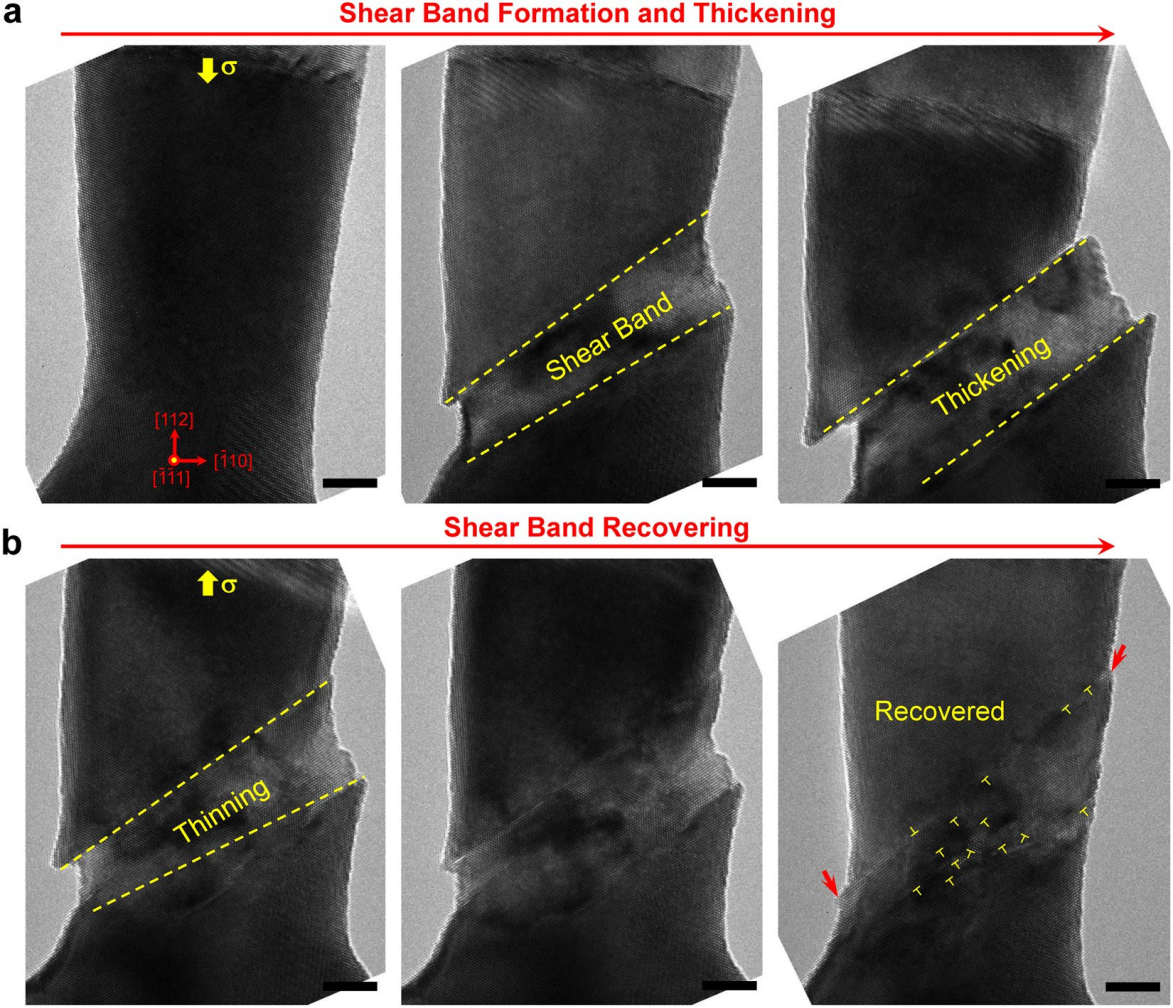
Recoverable shear band in a [112]-W nanowire in loading cycle. The nanowire diameter is about 22 nm. (**a**) A shear band with surface steps forms under the compressive loading. The thickness of the shear band is about 8.5 nm before the loading is reversed. (**b**) The shear band is gradually recovered via the thinning of shear band and the shrunk of surface steps under reversed loading. However, a lot of dislocations are left in the crystal lattice after the loading cycle, most of which are locating at the shear band interface. All scale bars are 5 nm.

In the loading-unloading cycles, shear band deformation is partially recoverable, as shown in Fig. 4. Under the compressive loading, large plasticity is achieved in W nanowire via the dislocation slip and shear band thickening, the thickness of which reaches ~8.5 nm (Fig. 4a) before the loading is reversed. Under the reversed (tensile) loading, plastic deformation of W nanowire is able to recover without fracture from the shear band (Fig. 4b). This recovering process is mediated by reverse slip of dislocations along the shear band interfaces, resulting in the gradual thinning of shear band and reduction of surface steps. The shear band interface moves gradually because it occurs by the motion of existing dislocations and no dislocation nucleation is required. Unlike the twinning-mediated deformation with full recoverability^23^, the shear band mediated plastic deformation cannot be completely reversed. Numerous defects, *i.e*. dislocations, are left in the lattice of shear band region after loading cycle (Fig. 4b). Surface steps left (marked out by the red arrows in Fig. 4b) after the cyclic deformation further indicate an incomplete recovery of shear band plasticity. Nonetheless, the partially recoverable plastic deformation of the shear band may still provide beneficial damage tolerance and fatigue resistance of W nanowire at the nanoscale.

## Discussion

Our findings indicate that the deformation, discrete thickening and recoverability of the shear band in metallic nanopillars are all controlled by the interface processes, through the interplay of dislocations and local strain concentration near the shear band interfaces. The formation and discrete thickening of shear band can contribute to repeating strain bursts and thus stress drops (*i.e*. softening) in the stress-strain curve of small crystals^18,19,28^, while the gradual accumulation of shear strain near the shear band interface is caused by the re-buildup of deformation stress. Moreover, our experiments also show that high density of dislocations can be stored in the BCC W nanowire after the initial yielding (Figs 1 and S2), in contrast to the recent observations in FCC and BCC sub-micro pillars fabricated by focus ion beam (FIB) cutting, in which mechanical annealing was observed throughout most of the deformation^4,18,24^. We postulate that the smaller size and clean structure of our samples are responsible for the difference between these observations and ours. In the FIBed samples, pre-existed lattice defects dominate the deformation and those defects can easily escape to the free surface, causing the mechanical annealing^4,18,24^. However, in our sample with smaller size and clean structure, dislocation nucleation plays a more significant role due to the lack of plastic carrier. Besides, the critical stress for the surface-induced nucleation and self-multiplication increases with decreasing sample diameter. This allows the stress to be raised to a higher value for nucleation to occur at multiple surface sites, before the sudden strain softening sets in. This difference may play a role in the observed different strengthening trends in small scale FCC and BCC single crystals^32^.

In summary, our TEM observations and atomistic simulations have shown that the surface structure of small BCC metals strongly affect the nucleation of dislocations, and the coupling between the stress concentration near the shear band and dislocation nucleation leads to discrete jumps of the shear band thickness. Part of the shear band plasticity is recoverable through reverse motion of dislocations. Our study advances the current understanding of free surface on the defect nucleation in small BCC metals and alloys. The interface controlled deformation and thickening mechanism of shear band may have general application in understanding the deformation morphologies of nanoscale specimens of other crystal structures.

## Materials and Methods

### ***In situ*** TEM nanomechanical testing

The bicrystal W nanowires were created by an *in situ* welding process inside a FEI Tecnai F30 field emission gun (FEG) TEM. A Nanofactory TEM-scanning tunneling microscope (STM) platform was used for sample fabrication and nanomechanical testing. Bulk polycrystalline W rods with the purity of 99.98 wt.% and diameter of 0.010 inch (ordered from ESPI Metals Inc.) were used in current experiments. The impurities in these W rods are: Cr (10 ppm), Ni (130 ppm), Fe (20 ppm), K (<20 ppm) and Cu (<20 ppm). Before experiments, a bulk W rod was fractured by a tungsten carbide plier to obtain the fresh fracture surface with nanoscale teeth. During experiments, a bulk polycrystalline W rod served as one end in the TEM-STM platform, while a W probe was used as the other end of the platform. The W probe was then driven by a pizeo-controller to contact with a nanotooth on the fracture surface of the W rod. During the welding process, a 2 V potential was applied between the W probe and a nanotooth, which welded the two tungsten crystals together at the moment of contact, forming a W nanowire. Usually, bicrystal W nanowires were obtained due to the misorientation between the W probe and nanotooth. *In situ* compression or tension testing was then performed at room temperature by moving the W probe forward or backward at a constant velocity of ~0.05 nm/s, giving a strain rate of about 10^−3^ s^−1^. The real-time deformation process was recorded by a CCD (charge-coupled device) camera at 2 frames per second.

### Nucleation Barrier Calculation

The dislocation nucleation barrier calculations were performed with the molecular dynamics simulation package MD++. The Ackland-Thetford-Finnis-Sinclair potential^29,30^ was adopted for BCC W, which could predict several important dislocation associated properties (such as Peierls Potential and the gamma surface) that are the most consistent to the DFT results though it cannot accurately reproduce the screw dislocation core structure in DFT^33^. First, a BCC W perfect single crystal was created with dimensions of: 14 $[\mathop{1}\limits^{-}10]$ × 10[112] × 12 $[\mathop{1}\limits^{-}\mathop{1}\limits^{-}1]$ (*x* × *y* × *z*). From this bulk configuration, the two {111} free surfaces were generated by extending the simulation cell in *z* direction (by 20%) with keeping the real coordinates of atoms. The same procedure can be applied to create the {110} open surfaces. It is worth to mention that the {111} surface (normal to *z* axis) has a zigzag profile (with three different layers), while the {110} surface appears atomically flat (with a single layer), see Supplementary Fig. S3. Next, a compressive strain along [112] direction (ε_yy_) was applied to the simulation cell. All other non-zero strain components (ε_xx_ and ε_zz_, due to the Poisson effect) were adjusted to make all stress components except σ_yy_ go to zero. An improved string method^30^ was used to search for the minimum energy path between state A (perfect crystal) and state B (containing a perfect dislocation loop) under different amount of normal strain ε_yy_ (compressive), for dislocation nucleating from $(\mathop{1}\limits^{-}\mathop{1}\limits^{-}1)$ and $(\mathop{1}\limits^{-}10)$ surfaces respectively (Supplementary Fig. S3a and b). An open visualization tool OVITO was employed to visualize the defects during the deformation^31^. Only atoms with the central symmetry deviation (CSD) parameter exceeding a threshold value are plotted to show the dislocation core (Fig. 2b,c). With the help of Dislocation Analysis (DXA), the dislocations were recognized as green lines with yellow arrows denoting the corresponding Burger vectors^32^. In addition, simulation snapshots were taken with the atoms colored by the CSD parameters, to illustrate the dislocation nucleation process on {110} and {111} surfaces respectively (Supplementary Fig. S5).

### Finite Element Simulation

The finite element simulations were performed by the commercial software ABAQUS. A kinked nano-pillar with an inclined section at 30 degree (the angle between [112] and [101]) was created, as shown in Supplementary Fig. S6a. The dimensions of the kinked pillar were set to 25 nm in diameter and 55 nm in height, such that they were in consistence with the experimental scales. The Young’s modulus of 400 GPa and Poisson ratio of 0.28 were used for tungsten. Two parallel rigid plates were created to clamp the sample at the top and bottom surfaces, and the friction coefficient of their contacting surfaces was set to 0.1. As we expected, we found the simulation results were insensitive to the specific value of the friction coefficient. Nonetheless, the friction coefficient should not be too small, in order to prevent from the sliding between the nanowire and the plates. The bottom plate was fixed, while a vertical displacement was applied on the upper plate to compress the pillar. The assembly was meshed with 12153 C3D4T elements, followed by a simulation that compressed the sample to an overall compressive strain of 12%, during which the geometric nonlinearity for large strain deformation was accounted for.

## Electronic supplementary material

Supplementary Information
