# Supplementary material for: Discrete shear band plasticity through dislocation activities in body-centered cubic tungsten nanowires
Source: Sci Rep. 2018 Mar 15;8:4574. doi: 10.1038/s41598-018-23015-z (PMC5854623; doi:10.1038/s41598-018-23015-z)
Supplement: Supplementary file 1 — Supplementary Information [file 41598_2018_23015_MOESM1_ESM.pdf]

Supporting Information for

Discrete shear band plasticity through dislocation activities in body-centered cubic tungsten nanowires

Jiangwei Wang[1,2,#,*], Yanming Wang[3,#], Wei Cai[4,*], Jixue Li[1], Ze Zhang[1], Scott X. Mao[2,*]

[1]Center of Electron Microscopy and State Key Laboratory of Silicon Materials, School of Materials Science and Engineering, Zhejiang University, Hangzhou 310027 China

[2]Department of Mechanical Engineering and Materials Science, University of Pittsburgh, Pittsburgh, Pennsylvania 15261, USA

[3]Department of Materials Science and Engineering, Stanford University, Stanford, California, 94305, USA

[4]Department of Mechanical Engineering, Stanford University, Stanford, California, 94305, USA

Corresponding authors: jiangwei_wang@zju.edu.cn; caiwei@stanford.edu; sxm2@pitt.edu.

[#] These authors contribute equally to this work.

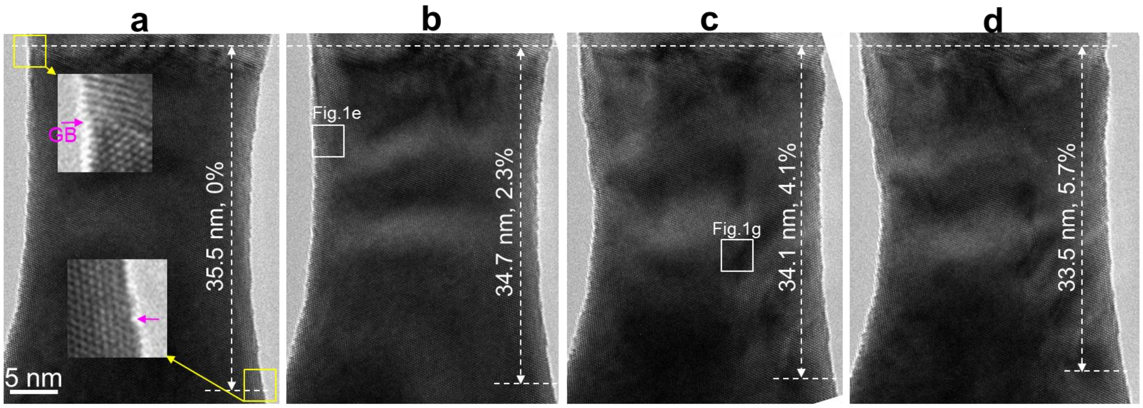

Supplementary Figure S1 Measurement of compressive strain during the deformation of W nanowire shown in Figure 1. (a) The morphology of intial nanowire. The insets show two fine structures on surface (the GB/surfcae intersection and a surfcae step) that were used as reference for the compressive strain measurement. (b) At the compresisve strain of 2.3%, the nanowire yields via the dislcoation nucleation from side surface. The dislcoation structure is presented in Figure 1e and 1f. (c) As the compressive strain increases (higher than ~4%), the dislocation mainly nucelate as dipoles from the viewing surface. Figure 1g shows an example of the dislocation dipole. (d) Further straining causes the nucleation of more dislcoations, which is detailed in Figure 1b.

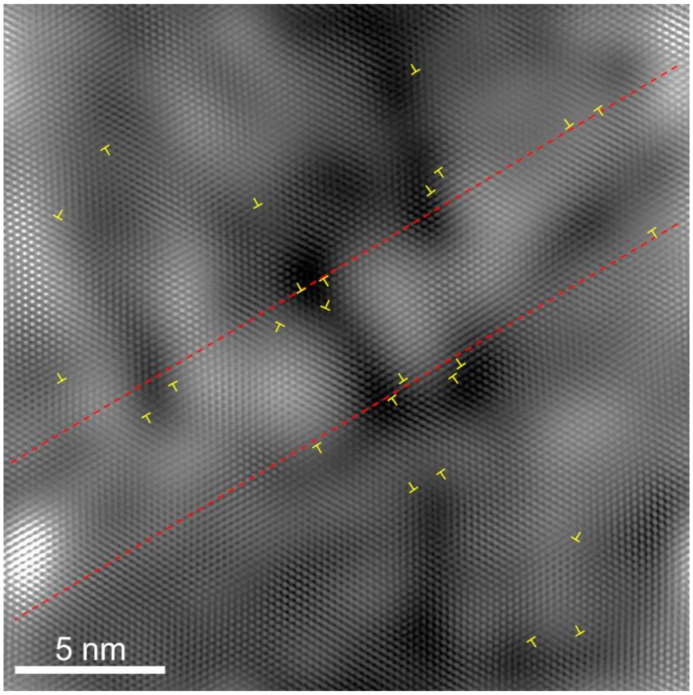

Supplementary Figure S2 Fourier-filtered HRTEM image of Figure 1c showing the distribution of dislocations after the shear band formation. The red dashed lines mark out the interfaces between the shear band and the surrounding crystals. The dislocation density is ~ 4×10^{16} m^{-2} and the compressive strain is ~ 11%.

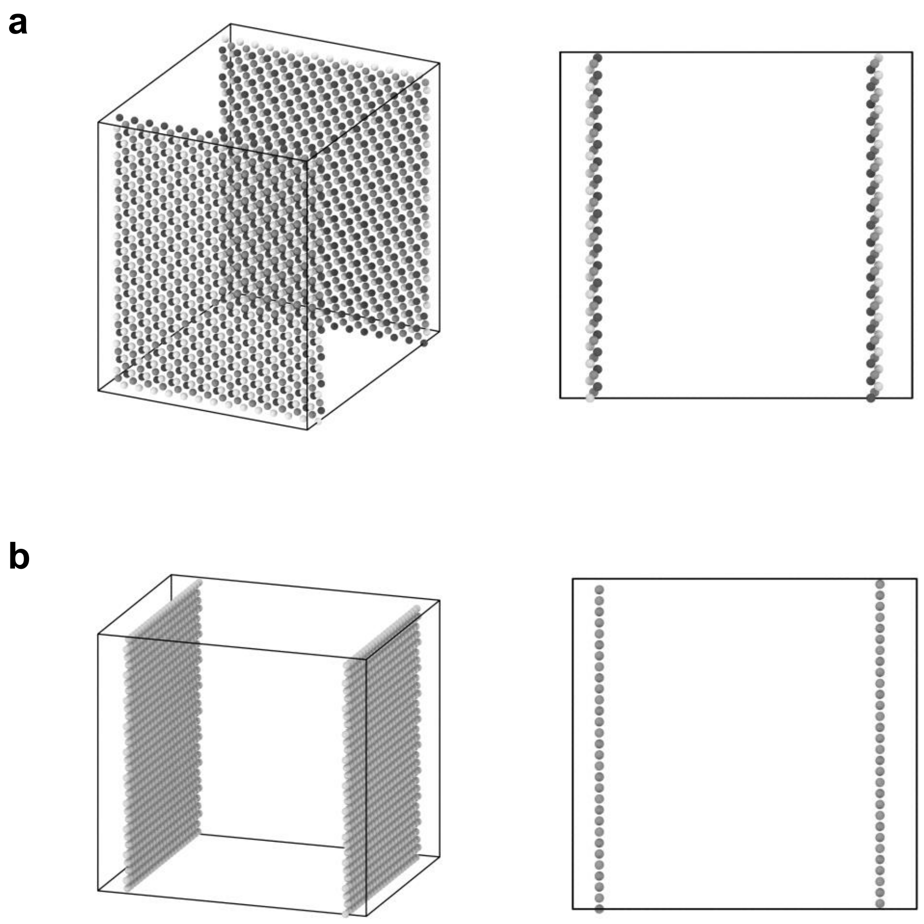

Supplementary Figure S3 3D and side views of the simulation cells with different surface orientations. (a) (111) surface with a zigzag profile involving three atomic. (b) $(1\bar{1}0)$ surface with an atomically-flat structure involving a single layer.

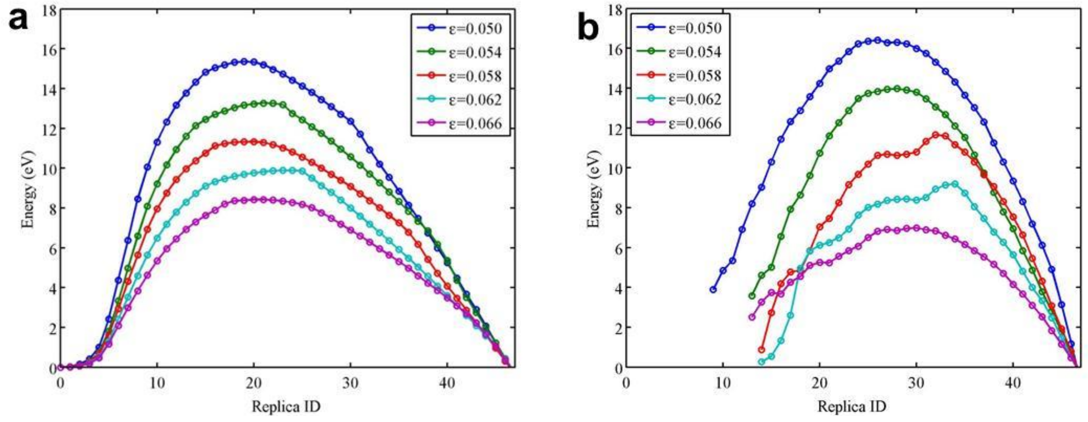

Supplementary Figure S4 Energy profiles for the surface nucleation of glide dislocation loop in W at different shear strains. (a) The energy profile for half loop nucleated from (111) surface at different amount of compressive strain ε_{yy}. (b) The energy profile for half loop nucleated from $(1\bar{1}0)$ surface at different amount of compressive strain ε_{yy}.

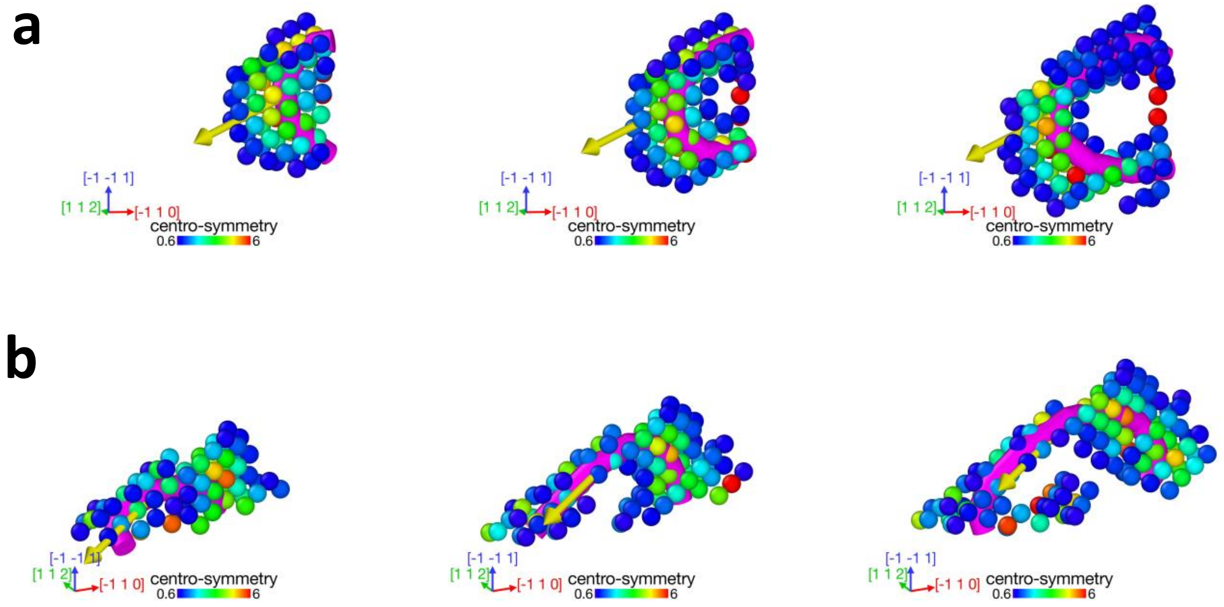

Supplementary Figure S5 Evolution of atom configurations (the atoms are colored based on the central symmetry parameter) during the dislocation nucleation process. (a) dislocation nucleation on $(\bar{1}10)$ surface. (b) dislocation nucleation on $(\bar{1}\bar{1}1)$ surface.

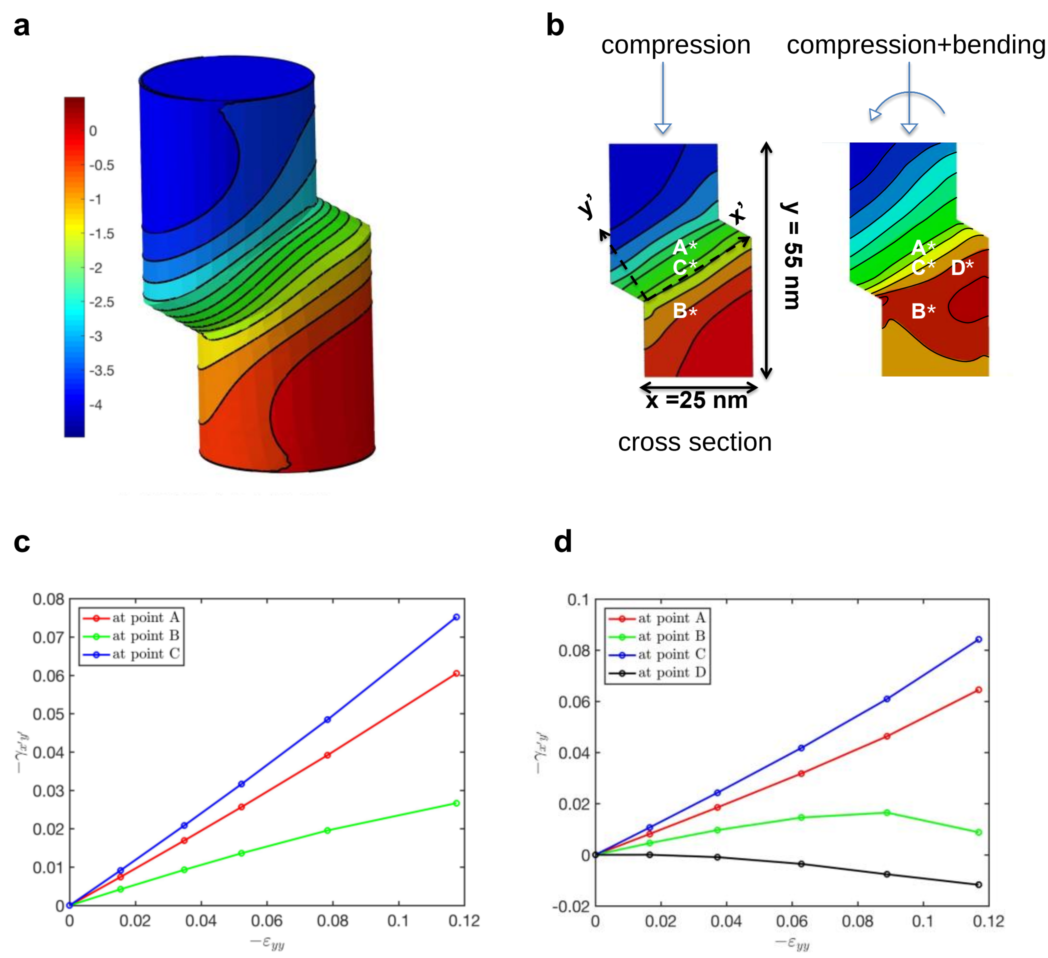

Supplementary Figure S6 Finite element simulation of W nanowire under [112] compression. (a) Iso-surface plots of the displacement field along *x'* direction in 3D view for the kinked pillar under compression. (b) Iso-surface plots of the displacement field along *x'* direction in cross sectional views for the kinked pillar under compression (left) and compression with bending (right). (c) The resolved shear strains on the slip plane ($-\gamma_{x'y'}$) are plotted as a function of compressive strain ($-\varepsilon_{yy}$) at point A (inside the shear band), point B (outside the shear band) and point C (near the shear band interface), for pillar under compression. (d) The shear strains on the slip plane ($-\gamma_{x'y'}$) are plotted as a function of compressive strain ($-\varepsilon_{yy}$) at point A, B, C and D for kinked pillar under compression with extra bending.
